# Supplementary material for: The effect of intrapartum prolonged oxygen exposure on fetal metabolic status: secondary analysis from a randomized controlled trial
Source: Front Endocrinol (Lausanne). 2023 Jun 27;14:1204956. doi: 10.3389/fendo.2023.1204956 (PMC10335765; doi:10.3389/fendo.2023.1204956)
Supplement: Supplementary file 3 [file DataSheet_3.zip › Result-X101SC21092977-Z01-J001-B1-42/2.MetAnnotation/KEGG/meta_pos.KEGG.Anno.pdf]

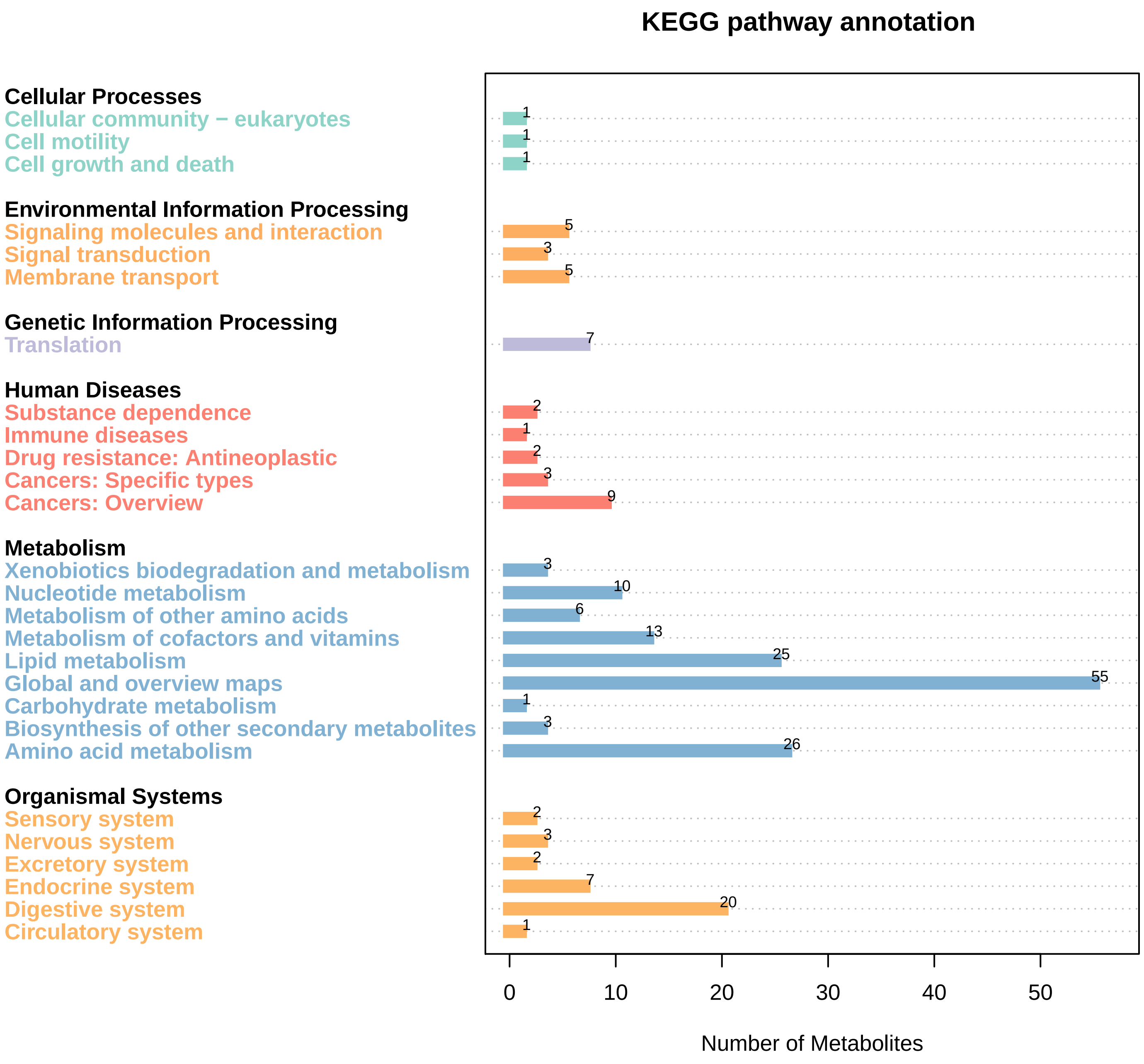
KEGG pathway annotation
Cellular Processes
Cellular community − eukaryotes
Cell motility
Cell growth and death
Environmental Information Processing
Signaling molecules and interaction
Signal transduction
Membrane transport
Genetic Information Processing
Translation
Human Diseases
Substance dependence
Immune diseases
Drug resistance: Antineoplastic
Cancers: Specific types
Cancers: Overview
Metabolism
Xenobiotics biodegradation and metabolism
Nucleotide metabolism
Metabolism of other amino acids
Metabolism of cofactors and vitamins
Lipid metabolism
Global and overview maps
Carbohydrate metabolism
Biosynthesis of other secondary metabolites
Amino acid metabolism
Organismal Systems
Sensory system
Nervous system
Excretory system
Endocrine system
Digestive system
Circulatory system
1
1
1
5
3
5
7
2
1
2
3
9
3
10
6
13
25
55
1
3
26
2
3
2
7
20
1
0
10
20
30
40
50
Number of Metabolites
